# Characterization of insulin-like-growth factor II (IGF II) mRNA positive hepatic altered foci and IGF II expression in hepatocellular carcinoma during diethylnitrosamine-induced hepatocarcinogenesis in rats

**DOI:** 10.1186/1477-3163-4-12

**Published:** 2005-08-10

**Authors:** Biswajit Mukherjee, Shampa Ghosh, Tanushree Das, Manika Doloi

**Affiliations:** 1 Department of Pharmaceutical Technology, Jadavpur University, Kolkata 700 032, India; 2 Department of Biochemistry and Nutrition, All India Institute of Hygiene and Public Health, C.R. Avenue, Kolkata 700 073, India

**Keywords:** IGF II, hepatocarcinogenesis, rat, mRNA, hepatic altered foci

## Abstract

**Background:**

Insulin-like-growth factor II (IGF II) has been implicated in the pathogenesis of neoplasm of different tissues, including liver of rats and men. This growth factor is believed to exert its effect during cellular proliferation. During the process of development of hepatocellular carcinoma (HCC), different hepatic altered foci appear. They are believed to be the putative precursors of HCC in rats and in men. Thus, to study the role of the gene in a defined model of hepatocarcinogenesis was the target to elucidate its role in various cancer phenotypes during the entire development stage of cancer, right from earlier preneoplastic lesions to HCC

**Methods:**

Antisense in situ hybridization technique was used here to characterize the type(s) of foci in which IGF II mRNA had expressed during the development of hepatocarcinogenesis-induced by diethylnitrosamine and promoted by phenobarbital in rats. Various focal lesions have been categorized depending on the stages and sizes along with IGF II expression patterns in them. Immunohistochemical detection for proliferating cell nuclear antigen (PCNA) was made to detect the role of the gene in preneoplastic and neoplastic cellular proliferation.

**Results:**

IGF II expression was located in the glycogen-storage acidophilic cell foci maximally followed by mixed cell lesions and the least in basophilic lesions. The expression of IGF II was found to be predominant in the HCC. The expression of gene was also located at the peripheral cells of spongiosis hepatis which are believed to be the precursor of ito cell carcinoma. It was noted that there is a direct correlation between IGF II expression and Immunohistochemical detection for PCNA.

**Conclusion:**

It may be concluded that IGF II gene expression plays an important role during the development of neoplasia and the gene expresses in the sequence of events leading from glycogen-rich-acidophilic lesions to glycogen poor basophilic lesions to HCC with an expression pattern of “high-low-high” in terms of degree of expression. Moreover, the essential role of the gene at the immediate initiation stage of carcinogenesis (first few weeks) and during HCC development cannot be ignored. Thus this expression can be used as a suitable marker for very early detection of the cancerous process and can save numbers of future cancer victims by very early detection of this disease.

## Background

Insulin-like-growth factor II (IGF II)^b ^[somatomedin A ] is a mitogenic polypeptide having structural similarity to proinsulin and insulin-like-growth factor I (IGF I) [somatomedin C] [1,2]. IGF II is known to be widely distributed in various fetal and neonatal human and rat tissues, including liver [3-5], but restricted in the adult rat tissues [6], except brain [7]. It has also been implicated in the pathogenesis of neoplasm of different tissues, including liver of rats and men [8-11]. The growth factor is believed to exert their effect during cellular proliferation through endocrine, autocrine and paracrine mechanisms [12]. In breast cancer epithelial cells, IGFII was reported to serve as autocrine growth stimulus and IGFII overexpression was suggested to be capable of mediating malignant progression [13]. Expression of IGFII in rodents remains high around birth and becomes undetectable within three weeks [14]. But the reactivation of this gene has been reported in variety of neoplasms including HCC in rats. IGF II expression has been shown to be reactivated during progression of neoplastic nodules to HCC [15]. IGFII reactivation was reported to be a common phenomenon in hepatocarcinogenesis irrespective of species and the process of hepatocarcinogenesis [11]. The reactivation of endogenous IGF II was suggested to exert a growth stimulatory effect via IGFI and IGFII/mannose 6 phosphate receptors [16]. It was stated that IGFII might trigger a proliferating signal through hybrid insulin/IGFI receptors stimulated by exogenous IGF [17]. Carcinogen treatment may generate IGFII microenvironment in preneoplastic foci that lead to selective growth advantage for this cell.

Chemical hepatocarcinogenesis is a multistep and complex process and is a favorite model in rat to study the mechanism of transformation of a normal cell to malignant population(s) [18]. During the process of development of HCC from the normal hapatocytes, hepatic altered foci appear. These foci are the putative precursors of HCC in rat, and probably in man [19,20]. Depending on the morphological and biochemical hepatocellular changes during the process of development from normal hepatocytes to HCC induced by various chemical carcinogens, radiation, hormones, chronic viral hepatitis and transgenic manipulation in rodents, three main altered hepatocellular lesions-the glycogenotic, mixed cell and basophilic cell lesions have been distinguished [21]. The predominant sequence of cellular changes starts with glycogenotic clear and acidophilic (smooth endoplasmic reticulum- rich) hepatocytes and progresses through intermediate phenotypes in mixed cell populations to glycogen-poor, homogeneously basophilic (ribosome -rich) cellular phenotypes prevailing in undifferentiated HCC.

Elevated levels of IGF II gene and protein expression have been found in a wide range of human and rodent tumors [8-10]. IGF II has been claimed to be one of the most important growth factors, playing role during the progression of neoplasm [22]. But the advent of IGF II gene expression during the development of neoplasia has remained to be elucidated. Antisense *in situ *hybridization technique has been used here to explore the type(s) of foci in which IGF II mRNA is expressed at the stages of neoplastic conversion of hepatocytes during diethylnitorsamine (DENA)-induced and phenobarbital promoted hepatocarcinogensis in Sprague-Dawley rats.

## Materials and methods

Experiments were conducted on the hepatic tissues of male Sprague-Dawley rats (purchased from the Indian Institute of Chemical Biology, Kolkata, India). The initial body weights of the animals were approximately 130 g. They were maintained under constant temperature (22°C) and humidity (55%RH) conditions and were fed basal diet [23] and had free access to water. Animals were divided in four groups-Group A (having 15 animals), group B, C and D (having 10 animals in each of the three groups). Group A animals were normal untreated animals. Group B, C and D animals were carcinogen treated groups. All carcinogen treated animals were treated with DENA, 200 mg/kg body weight, intraperitoneally at the start of the experiment, i.e., day zero. Group C and D animals were promoted with 0.05 % phenobarbital in basal diet [24]. Group B animals were sacrificed at the 8^th ^week to study earlier preneoplastic lesions, group B animals were sacrificed at 22^nd ^weeks to study mixed and basophilic lesions and group C animals were sacrificed at 36^th ^week as HCC development are reported to take place between 32–36 weeks [25]. In each case five normal control animals were sacrificed with the respective treated groups B, C and D for comparison. All animals were unfed for 12 h before they were sacrificed by cervical dislocation and the animals were sacrificed between 9 and 10 a.m. The livers were removed, sliced (5–10 mm thick) and were snap-frozen in isopentane precooled with liquid nitrogen at – 150°C. The tissues were stored at – 80°C until further used.

Serial sections were prepared from the frozen materials with a Reichert-Jung microtome. They were studied histochemically by periodic acid Schiff reaction, toluidine blue and hematoxylin-eosin to detect different hepatic altered foci (26, 27). Preneoplastic lesions were eventually divided into three categories in accordance with their respective size and the total area of the occupied liver parenchyma (<1 mm, >1 mm-<3 mm, >3 mm) as described previously [28]. These serial sections were used for *in situ *hybridization with sense and anitsense digoxigenin labeled mRNA probes of IGF II and immunohistochemical reaction for *proliferating cell nuclear antigen *(PCNA).

### In vitro transcription of Digoxigenin (DIG)-labeled sense and antisense IGF II mRNA

**Figure 1:**
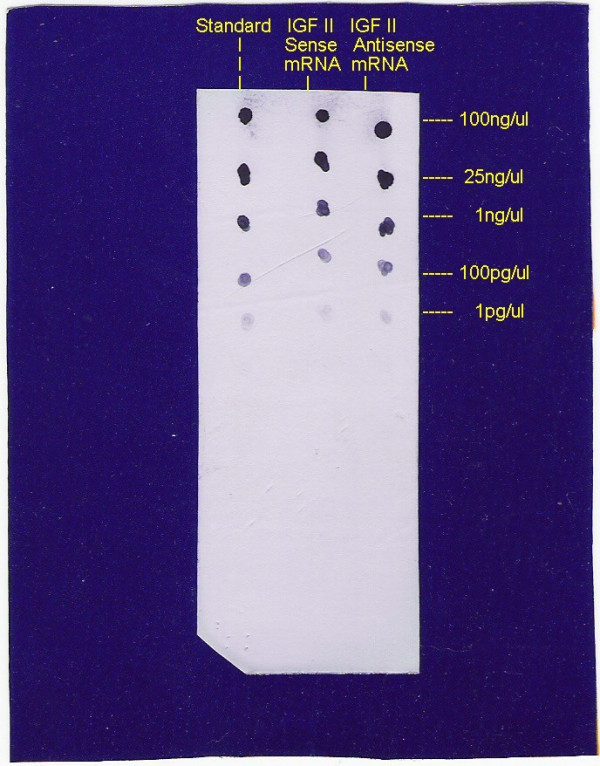
Quantification of DIG-labeled sense and antisense IGFII mRNA by spot test against standard labeled mRNA (Boehringer-Mannheim RNA detection kit).

**Figure 2:**
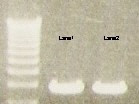
Detection of DIG-labeled IGFII sense and antisense mRNA (860 bp) using gel electrophoresis. Lane 1; DIG-labeled sense IGFII mRNA, Lane 2; DIG-labeled antisense IGFII mRNA.

A 860 base pair (bp) coding region fragment from rIGF II cDNA extended between EcoRI- EcoRI ploylinker site of pGEM-4 vector (Source: H. Hacker, DKFZ, Germany, obtained from A Riccio, Universita degli studi di Napoli Federico II, Napoli, Italy) was transformed into XL1-blue *E.coli *competent cells. The plasmid was also religated and propagated in the competent cells to obtain the opposite orientation of the 860 bp coding fragment of rIGF II cDNA. SP6-RNA polymerase was used to generate both sense and anitsense riboprobes from Xba I linearized plasmids of the opposite orientation. *In vitro *transcription was conducted, using digoxigenin (DIG)-RNA labeling Kit from Boehringer-Mannheim, Mannheim, Germany. The concentrations of the sense and antisense probes were estimated by using Nucleic Acid Detection Kit (Boehringer- Mannheim, Mannheim, Germany). The method is based upon the comparison between the colour intensities of slot blots and DIG labeled standard (Boehringer- Mannheim, Mannheim, Germany) (Figure 1) and the experimental riboprobes. By electrophoresing the probes on formaldehyde containing 1% agarose gel the purity of the labeled probes were checked (Figure 2).

### Detection of IGF II mRNA by *In situ *hybridization

*In situ *hybridization was conducted on 6 μm cryosections of the liver samples by using a modified procedure of Braissant and Walhi [29]. The cryosections were first fixed at 50°C for 30 seconds on a hot plate and delipidified with chloroform for 5 min whenever necessary. The sections were then fixed in 4% paraformaldehyde in phosphate buffer saline, pH 7.4, (PBS) for 1 hr. The slides were then treated with PBS containing active DEPC (diethyl pyrocarbonate) for 30 min followed by a wash of 4 × SSC of 15 min. Prehybridization was conducted using hybridization buffer (mentioned below) except dextran sulphate and Denhardt's solution for 1 hr. at 52°C. This was followed by hybridization at 50°C overnight in a humid chamber. Every 10 ml of hybridization buffer contained 5 ml deionized formamide, 2.4 ml DEPC treated water, 2 ml 20 × SSC, pH 7.0, 1 g dextran sulphate, 200 μl 50 × Denhardt's solution, denatured yeast t-RNA (10 mg/ml) 400 μl and denatured salmon sperm DNA (10 mg/ml) μl along with respective probes denatured by heating at 80°C for 3 min., followed by immediate cooling on ice. After hybridization, sections were washed in 2 × SSC at room temperature (RT) for 30 min, then again with 2 × SSC at 60°C, for 30 min. This was followed by 1 × SSC for 30 min and 0.1 × SSC for 1 hr, at 60°C with a frequent moderate shaking. After the stringency wash, the slides were incubated with Tris-NaCl buffer, pH 7.5 containing 0.5% blocking agent (Boehringer-Mannheim, Mannheim, Germany) at RT on a shaking platform with a low speed. Sections were then incubated with anti-DIG-polyclonal antibody conjugated with alkaline phosphatase (Boehringer-Mannheim, Mannheim, Germany) (1:5000) in Tris-NaC1 buffer, pH 7.5 for 2 hr at RT on the shaking platform with a low speed. After incubation, sections were washed with washing buffer (Tris-NaC1 buffer, pH 7.5, containing 0.3% tween 20) for 10 min at RT. This was followed by two 15 min washes with Tris-NaC1 buffer, pH 7.5. the section were equilibrated with detection buffer (100 mM Tris-HC1, 100 mM NaC1 and 50 mM MgC1_2_,6H_2_O), pH 9.5 for 5 min and staining was done using nitroblue tetrazolium/ bromochloroindolylphosphate (NBT/BCIP) in detection buffer. Counter staining was also done occasionally.

### Immunohistochemical detection for *proliferating cell nuclear antigen*

PCNA was demonstrated by following the methods of Enzmann et al. [30] and Bannasch et al [31]. Briefly 6 μm cryostat sections were air-dried after fixation in acetone for 10 min followed by a treatment with 2% H_2_O_2 _in methanol to block endogenous peroxidases. Then the sample was incubated in 3% bovine serum albumin for 30 min to prevent unspecific reactions followed by incubation with mouse monoclonal antibody to PCNA, diluted 1:1600 (PC10 Dako), overnight at 4°C. PCNA positive nuclei were identified by using alkaline phosphatase labeled streptavidine-biotin complex (Dako LASB2 Kit, AP). Then the sections were stained with new-fuschin and Myer's haemetoxylin.

IGF II mRNA positive foci were estimated in randomly selected areas of each section containing hepatic altered foci from experimental animals. The results were statistically evaluated using ANOVA followed by Fisher's exact Test otherwise mentioned.

## Results

**Figure 3:**
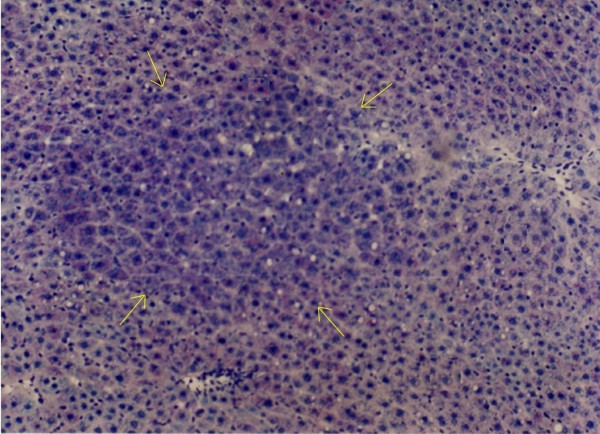
Glycogen storage foci (shown by yellow arrows) in rat hepatic tissue with Periodic Acid Schiff reaction, ×100.

**Figure 4:**
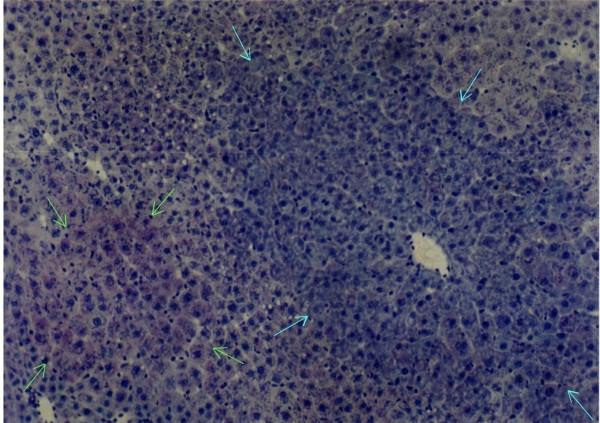
Mixed cell focal lesion (shown by green arrows) and basophilic lesion (shown by blue arrows) in DENA treated rat liver, ×100.

The morphometric determination of the focal lesions showed that incidences of preneoplastic lesions (i.e. glycogen storage foci/acidophilic foci [Figure 3], mixed cell focal lesions and basophilic focal lesions [Figure 4] were 100% in group B, C and D animals. The numerical densities and size distributions of preneoplastic lesions were depicted in Table 1. Average numerical density was found to be maximum (1498 ± 33 per cm^3 ^of hepatic tissue) in case of group D animals, which was followed by group C (968 ± 16 per cm^3 ^of hepatic tissue) and group B (412 ± 28 per cm^3 ^of hepatic tissue) animals. The preneoplastic lesions were categorized with respect to their sizes and their areas of the occupied liver parenchyma. It was observed that group B animals in which carcinogenesis had been initiated with DENA and animals were sacrificed after 8 weeks, had 81% focal lesions having size less than 1 mm i.e. smaller lesions and 19% focal lesions were in the size range of 1 mm-3 mm. Group C had 52% lesions less than 1 mm. 44% lesions were between 1 mm and 3 mm and sizes of 4% lesions were greater than 3 mm. In group D animals, seven animals out of ten were found to develop HCC (Table 2) and they had more intermediate (1 mm-3 mm) and large (greater than 3 mm) lesions as compared to smaller lesions.

**Table 1:** Incidences, numbers and size distributions of preneoplastic lesions (glycogen-storage, mixed cell and basophilic type) in the hepatic tissues of male Sprague-Dawley rats in different experimental groups (B, C and D).

Group	No. of rats with preneoplastic lesions/Total numbers of rats	Incidences (%)	Numerical densities of preneoplastic lesions^a,b^	Size distributions of preneoplastic lesions (% of total numbers)		
				
				<1 mm	>1 mm-<3 mm	>3 mm
B	10/10	100	412 ± 28	81	19	-
C	10/10	100	968 ± 16	52	44	4
D	10/10	100	1498 ± 33	20	48	32

^a ^Numerical density, expressed in number per cm^3 ^of hepatic tissue

^b ^Each value represent mean of 10 rats per group ± SD

**Table 2:** Expression of IGF II gene in different types of preneoplastic hepatic altered focal lesions (HAF) as well as carcinoma in hepatic tissues of the experimental animals (group B, C and D)

Types of HAF which expressed IGF II gene	Numbers of rats with HAF and/or HCC per total no of animals	Number of IGF II expressed lesions/100 respective HAF lesions
Glycogen-storage focal lesion	10/10	98 ± 2.98^a,^*
Mixed cell focal lesion	10/10	74 ± 8.26^a,^*
Basophilic cell focal lesion	10/10	05 ± 2.68^a,NS^
Hepatocellular carcinoma	7/10	95 ± 4.02%^b,^*

^a ^Mean ± SD (n = 10)

^b ^Mean ± SD (n = 7)

* P < 0.001

NS- Non-significant

**Figure 5:**
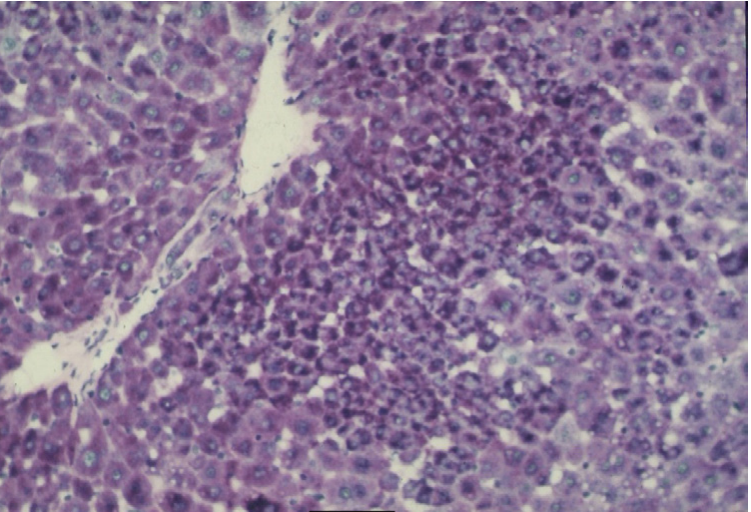
Hepatic section of DENA treated experimental animals showing glycogen storage foci with PAS staining, ×100.

**Figure 6:**
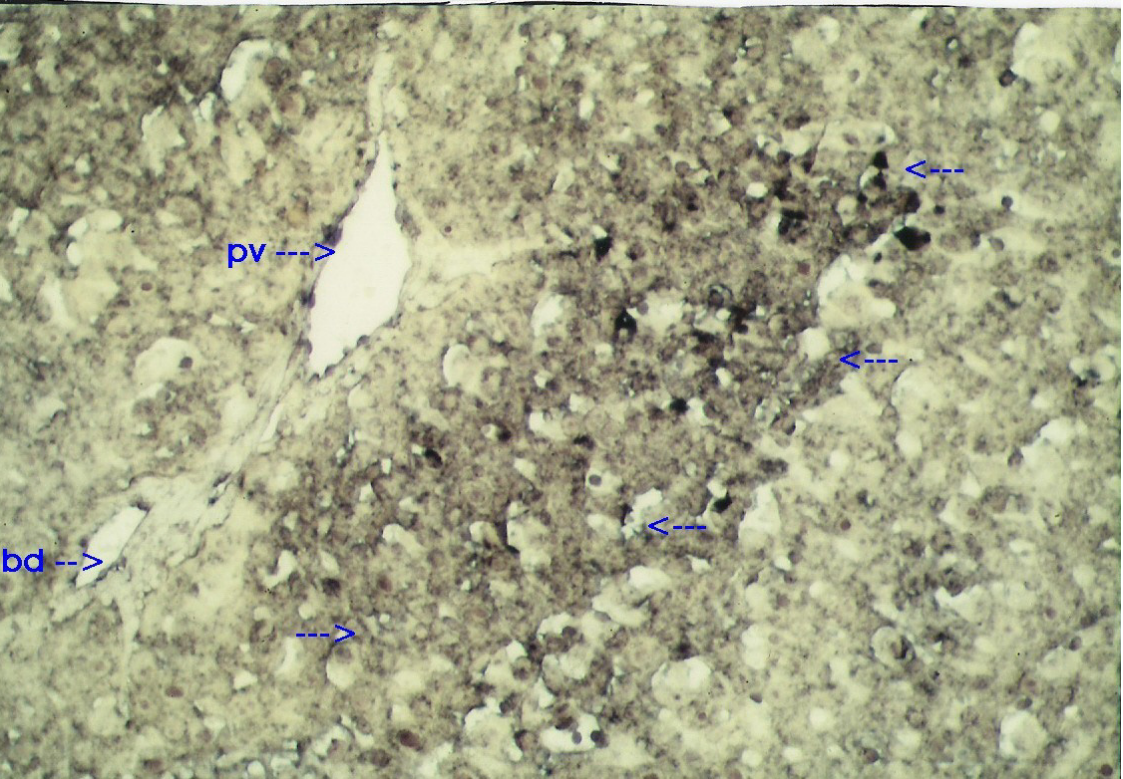
IGFII mRNA expressed glycogen storage lesion detected with Digoxigenin-labeled antisense IGFII, mRNA ×100.

**Figure 7:**
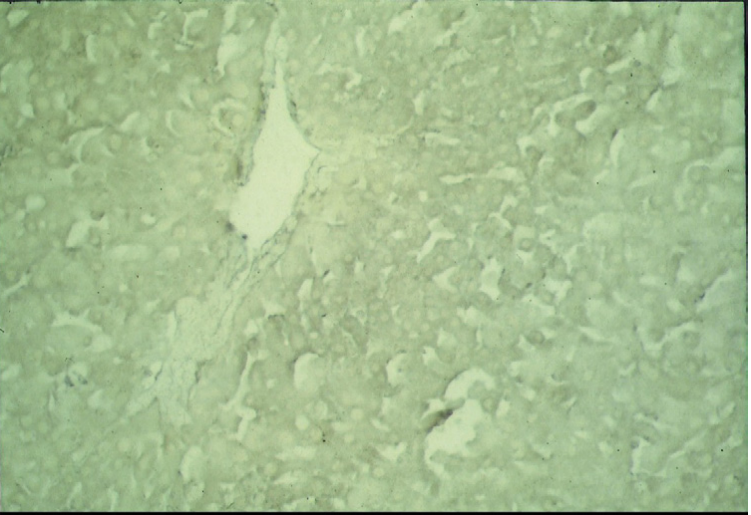
Glycogen storage lesion shown after Digoxigenin-labeled sense IGFII mRNA treatment during in situ hybridization method ×100. Pv- portal vein. Bd – bile duct.

**Figure 8:**
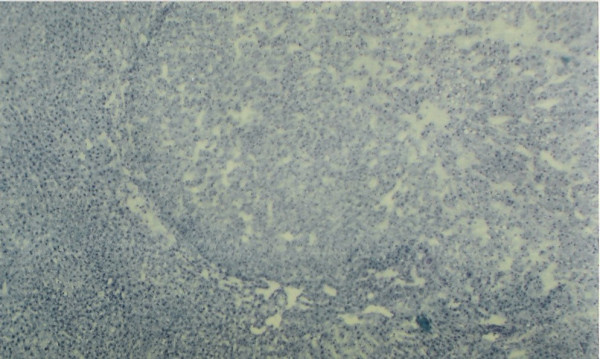
Section of hepatic tumor (shown by arrows) in HCC; using toluedine blue ×40.

**Figure 9:**
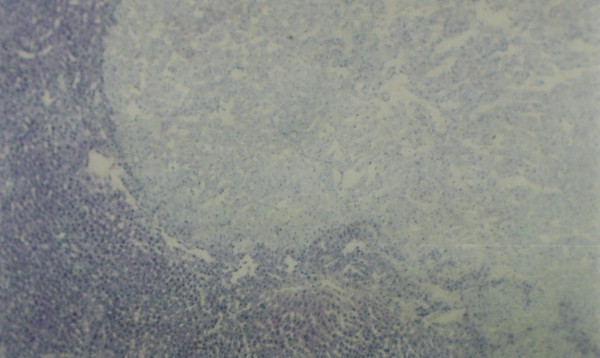
Section of hepatic tumor (shown by arrows) in HCC; using PAS reaction ×40.

**Figure 10:**
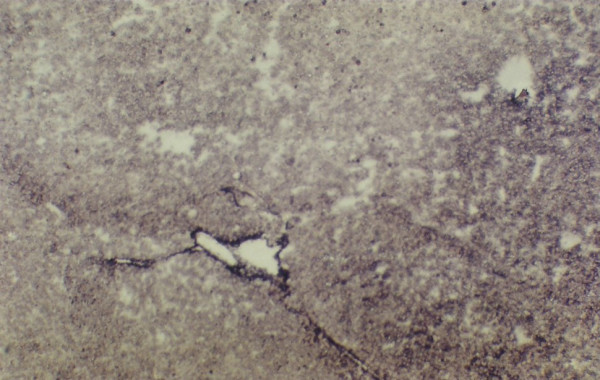
Section of hepatic tumor (shown by arrows) in HCC; IGFII mRNA expression detected with DIG-labeled IGFII antisense mRNA in tumor as well as peripheral tissue. ×40.

**Figure 11:**
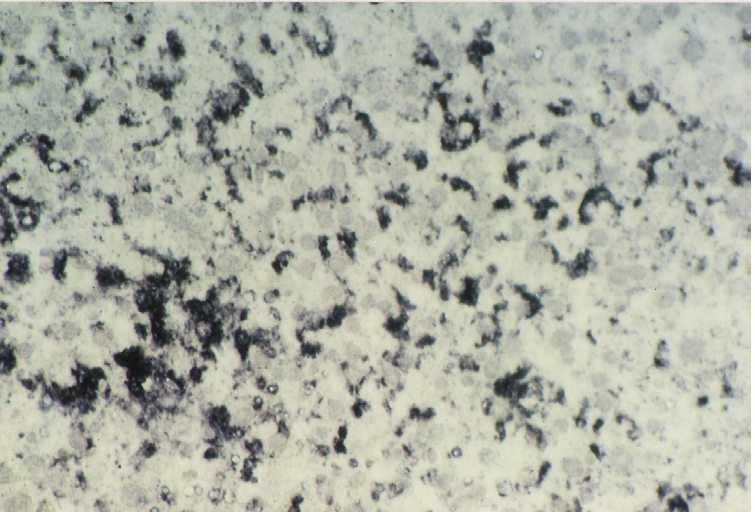
IGFII mRNA expression in hepatocytes in tumor area during HCC in experimental animals (see protocols) ×97.5.

When IGFII gene expressed, preneoplastic lesions were compared to the various type of hepatic altered foci (HAF) such as glycogen storage focal lesions, mixed cell focal lesions, and basophilic focal lesions, it was found that 98% glycogen storage focal lesions expressed IGFII [Figures 5, 6, 7]. Again 74% of the mixed cell focal lesions expressed IGFII where once again mainly glycogen storage hepatocytes were found to express IGFII not the basophilic cells. Very few basophilic cell focal lesions with IGFII gene expression were detected. In those lesions, there were, some glycogen containing cells (as seen with PAS staining). As the lesions containing predominantly basophilic cells, they were categorized under basophilic lesions. Thus it may be believed that glycogen storage cells showed IGFII expression in those basophilic lesions. Only 5% of the basophilic hepatic altered foci were found to express IGFII although the results are statistically non-significant. As described earlier in group D animals, seven out of ten animals were found to develop HCC and they had some high expression in the peripheral hepatocytes of tumor. There was also some expression within the tumor areas [Figures 8, 9, 10, 11].

Hepatocytes proliferation was established by demonstration of PCNA (Proliferating Cell Nuclear Antigen). PCNA positive hepatocytes and non-hepatocytes were detected in carcinogen treated groups as well as normal untreated groups. Five fold higher numerical value of PCNA positive cells was detected in the carcinogen treated animals as compared to normal untreated animals (Table 3) and PCNA positive cells in carcinogen treated animals were mostly (about 85%) confined to lesion area. Interestingly the counts were greater in glycogen storage lesions as well as in HCC (data not shown). This indicates that proliferation was higher during the early stage i.e. at the immediate post initiation stage as well as in HCC.

**Table 3:** Number of PCNA (Proliferating Cell Nuclear Antigen) positive liver cells per 10^3 ^cells

Labeling Index^a^		
**Group**	**HAF Hepatocytes**	**Non focal-hepatocytes**
Carcinogen-treated	667 ± 28*	122 ± 17*
Normal Control	132 ± 12	88 ± 14

^a ^results show mean ± SD (n = 10)

* P < 0.001 as compared to normal control animals, assessed by Student's *t*-test

**Figure 12:**
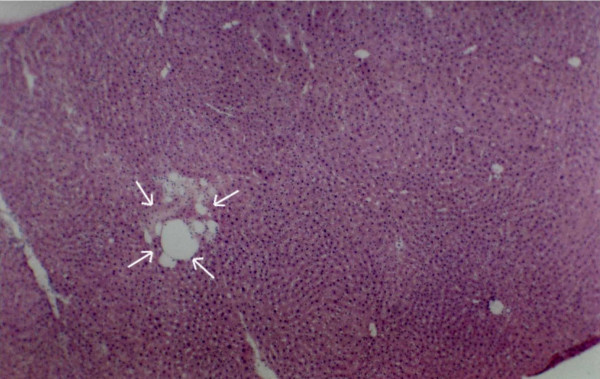
Serial sections of experimental rat hepatic tissue showing spongiosis hepatis with haematoxylin and eosin (shown by white arrows) ×40.

**Figure 13:**
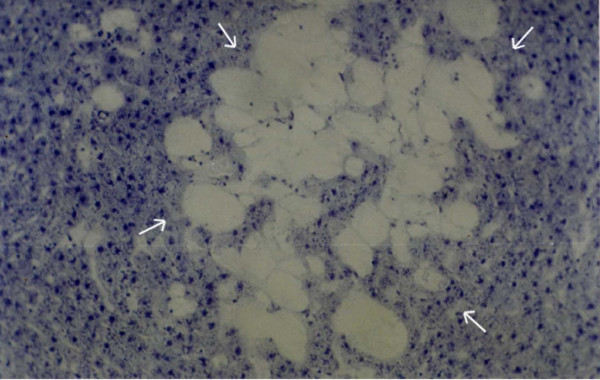
Section of experimental rat hepatic tissue showing spongiosis hepatis with toluedine blue ×120.

**Figure 14:**
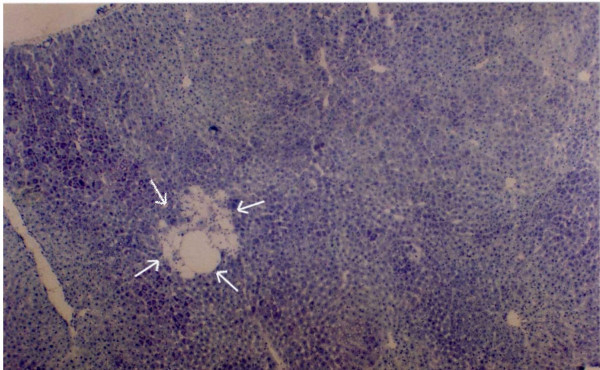
Section of experimental rat hepatic tissue showing spongiosis hepatis with PAS reaction) ×40.

**Figure 15:**
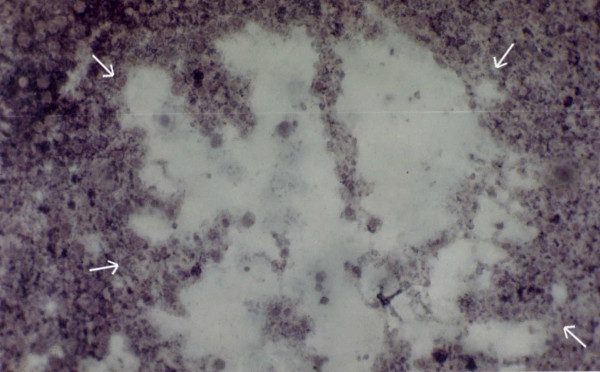
IGFII mRNA expression detected by DIG- labeled antisense IGFII mRNA in the peripheral cells of the spongiocyst hepatis ×120.

In the hepatic tissue of group D animals some scattered spongiotic pericytoma have been noticed. This spongiotic pericytoma are also believed to be the precursor of perisinusoidal cell sarcomas [32]. They were very few in numbers but were found in the hepatic tissue of all the animals in group D. But they were not found in every tissue slide of the same animals. When IGFII expression of the subsequent slides were studied it was observed that IGFII gene did not express in the area of spongiotic hepatis. But high IGFII expression was noticed at the peripheral hepatocytes [Figures 12, 13, 14, 15]. PCNA positive cells were also detected predominantly in the peripheral cells of spongiosis hepatis.

## Discussion

A predominant sequence of cellular changes starting from the appearance of glycogenotic foci to the glycogen-poor basophilic foci, leading to HCC via various mixed cell populations, has been observed in rodents during the development of HCC induced by various chemical carcinogens, hormones, radiation, chronic viral infection and transgenic manipulation [19,20]. During this process several phenotypic changes appear which suggest a progressive cellular dedifferentiation.

The DIG detection system used here has been claimed to offer a much higher sensitivity and detectability than radioactive detection systems [33,34]. Using this system, we tried to localize the IGF II gene expression in different type(s) of foci during DENA-induced hepatocarcinogenesis. Predominant expression of the gene was located in hepatic altered glycogen-storage foci which has been claimed to be early preneoplastic lesions [32] along with some expressions in foci of mixed cell populations. High expression was also detected in HCC. It was higher at the periphery as well as the glycogen storage cells of the tumor. This observation is supported by the observation of Sohda *et.al *who demonstrated that the localization of IGF II mRNA in areas consisting of less differentiated tumor were present at the periphery of the tumour nest [35]. Higher expression was detected in the foci of glycogen storage altered hepatocytes as compared to the tumor cells. This suggests that there is a differential expression of IGF II mRNA during the development of preneoplasia to neoplasia. Fetal transcripts of IGF II express high in man and rodent liver cancer [6]. In human, fetal transcripts express highly in the HCC, suggesting that IGF II-expression is late event during the development of liver cancer [36]. On the other hand, an inverse correlation was observed between IGF II-expression and cellular differentiation in rat [37]. Again, it has been found that the IGF II expression is predominant at the fetal and neonatal life of rat and it declines with the days after birth when IGF I is found to be increased and believed to take up the role of IGF II [6]. Simultaneously increased pattern of expressions of IGF I and TGFα, during some preneoplastic and neoplastic conditions [35] also suggests that the IGF I and TGFα may counterbalance the activity of IGF II.

Amongst the various methods available, two important methods of studying cancer cell proliferation are measuring the rate of DNA synthesis and the immunohistochemical reaction for *proliferating cell nuclear antigen *(PCNA) [30,31]. Demonstration of PCNA has been reported to be a valuable non-tracer method for the detection of proliferating cells. Report suggests the method is used to detect proliferating cells in S phase, G2 and late G1 phases of the cell cycles [32]. PCNA positive liver cell counts were found to be significantly higher (p < 0.001) in this study in carcinogen treated animals as compared to normal control animals. Higher PCNA labeling index were noticed in IGF II expressed lesions (mostly glycogen-rich lesions) as compared to the other IGF II non-expressed or little-expressed hepatic lesions (mixed cells and basophilic cell lesions) in hepatic areas studied. This suggests that the IGF II expressed cells in all the lesions are more proliferating in nature as compared to the other focal lesions and of course the extra-focal hepatic areas. The labeling index was found to be higher again in HCC. This indicates that cancer development is faster during glycogen-rich focal development (they predominantly expressed IGF II) and during the stage of HCC.

Spongiosis hepatis have been found interestingly only in-group C animals (in six out of ten animals). They were found to be very scattered in nature. In whole observation they were only 18 in numbers (in 50 different hepatic tissue samples of the seven animals of group C). Thus it seems that they appear in progression phase of cancerous process, at least during DENA-induced hepatocarcinogenesis. Spongiosis hepatis also described as spongiotic pericytoma are believed to be the possible precursor of malignant tumors [32]. It has also been classified as perisinusoidal (ito) cell sarcomas [33]. Lesions classified as spongiosis hepatis have been reported to progress to pericytomas which have been related to perisinusoidal cells (ito cells). Thus the study of these cells may help to understand neoplastic transformation of them. In present study efforts have been made to characterize various preneoplastic hepatic altered foci and hepatic cellular carcinoma along with spongiosis hepatis. Spongiosis hepatis have been detected using haematoxylin and eosin, toluidine blue and PAS staining. Consecutive sections were compared with the IGFII expressions and it has been observed that the predominant expression of IGFII were noticed in the peripheral hepatocytes but not in the holes of the spongiotic formations which are believed to be filled with a finely flocculent material that shows metachromasia (Acid mucopolysaccharide deposit) [32]. The IGFII expression seems to be dominant more in the peripheral hepatocytes of the spongiocyst hepatis. PCNA positive cells were also detected in the surrounding areas where the IGFII expressions were noticed. Again IGFII expression has been claimed to be associated with the development of hepatocellular carcinoma (HCC) by several authors [34,35]. Thus the study further supports that the spongiosis hepatis are possibly the origin of malignant tumor [32]

The claims related to an amount of over expression of IGF II gene in liver tumors and pre-neoplastic hepatic lesions during hepatocarcinogenesis in animal models and in human HCC were highly variable. A possible involvement of an IGF II autocrine loop in the pathogenesis of hepatic preneoplastic and neoplastic lesions have been linked with the association of allelic imbalance with an increased expression of the IGF II gene in pre-cancerous lesions [36,42].

The work has demonstrated that the allelic imbalance of the IGF II gene expression was seen in the early pre-cancerous lesions during hepatocarcinogenesis; but was not observed in well and moderately differentiated HCC.

Thus, some workers considered IGF II gene expression as an early event. Mixed reports are found which claimed the expression of IGFII either in preneoplasia [37,38] or during HCC [34,35]. The over expression of IGF II in HCC has been claimed to be associated with re-expression pattern of IGF II transcript occurring through activation of fetal promoters p2 – p4 with a loss of activity of the adult promoter p1 [36]. Interestingly, the over expression of IGF II was found in the preneoplastic glycogen storage focal lesions maximally as compared to that in the basophilic lesions.

In mixed cell focal lesions as well as basophilic lesions, the expressions were low and in the mixed cell focal lesions the expression was particularly confined to the glycogen rich cells. When the expression was predominant almost throughout a lesion, this has been considered as an IGF II expressed lesion in this study. So, the trend of IGF II over expression in our study was in the sequence of "high-low-high" in "glycogen storage foci – mixed cell foci – basophilic lesion – HCC". This suggests that the IGF II over expression is not only the early event, but the late event too. The gene expression amongst others appears to be responsible for cancer initiation process as well as in the progression of HCC. But its role during the progression from preneoplasia to neoplasia remains to be elucidated. Thus this gene over expression or may be the protein over expression can be used to detect the cancer at the early stage or at HCC and hence it may be considered as a future diagnostic means for detection of hepatic cancer and its stages and IGFII gene 33 expression could be considered as one of the best positive markers for early detection of putative preneoplastic cell as well as HCC in chemically induced hepatocarcinogenesis. Further investigation of this gene expression is warranted in the interest of better cancer detection. Thus this may lead to better cancer prevention. Again in contrast to many other gene or protein expressions IGFII has particular advantage for its use in screening of potential carcinogens and promoters since it is not expressed in lesions with some non-xenotoxic chemicals [39]. Further IGFII gene was reported to express at high levels in both spontaneous [40] and induced [41] hepatic lesions at the early stages of development [37,38] and in HCC [32,33].

## References

[B1] Daughaday WH, Rotwein P (1989). Insulin-like-growth factors I and II peptide, messenger ribonucleic acid and gene structures, serum and tissue concentrations. Endocrinol Rev.

[B2] Rechler MM, Nissley SP (1985). The nature and regulation of the receptors for insulin-like-growth factors. Ann Rev Physiol.

[B3] Brown AL, Graham DE, Nissley SP, Hill DJ, Strain AJ, Rechler MM (1986). Developmental regulation of insulin like growth factor II mRNA in different rat tissues. J Biol Chem.

[B4] Humbel RE (1990). Insulin like growth factor I and II. Eur J Bioche.

[B5] Cariani E, Lasserre C, Seurin D, Hamelin B, Kemeny F, Franco D, Czech MP, Ullrich A, Brechot C (1998). Differential expression of insulin like growth factor II mRNA in human primary liver cancer, benign tumors and liver cirrhosis. Cancer Res.

[B6] Moses AC, Nissley SP, Short PA, Rechler MM, White RM, Knight AB, Higa OZ (1998). Increased level of multiplication-stimulating activity or insulin-like-growth factor in fetal rat serum. Proc Natl Acad Sci USA.

[B7] Soares MB, Ishii DN, Efsratiadis A (1985). Development and tissue specific expression of transcripts related to the rat insulin-like-growth factor II mRNA. Nucleic Acid Re.

[B8] Ueno T, Takahashi K, Tetsuya T, Matsuguchi T, Ikejiri K, Endo H, Yamamato M (1988). Reactivation of rat insulin like growth factor II gene during hepatocarcinogenesis. Carcinogensis.

[B9] Li SL, Xu ZDX, Kimura G, Sun Y, Kawachi MH, Wilson TG, Wilczynk S, Yamaguchi YE (1998). Expression of insulin like growth factor (IGF)-II in human prostate, breast, bladder and paraganglioma tumors. Cell Tissue Res.

[B10] Uchida K, Kondo M, Takeda S, Osada H, Takahashi T, Nakado A, Takahashi T (1997). Altered transcriptional regulation of the insulin like growth factor 2 gene in human hepatocellular carcinoma. Mol Carcinogensis.

[B11] Schirmacher P, Held WA, Yang D, Chisari FV, Rustum Y, Rogler C (1992). Reactivation of insulin like growth factor II during hepatocarcinogensis in transgenic mice suggests a role of malignant growth. Cancer Res.

[B12] Stewart C, Rotwein P (1996). Growth, differentiation and survival; multiple physiological functions for insulin like growth factors. Physiol Rev.

[B13] Cullen KJ, Smith HS, Hills, Lippman ME (1991). Growth factor messenger RNA expression by human breast fibroblast from benign and malignant lesions. Cancer research.

[B14] Zapf J, Schoenle E, Froeasch ER (1978). Effect of insulin-like growth factor I and II: Some biological actions and receptors binding characteristics of two purified constituents of nonsuppressible insulin-like activity in human serum. Eur J Biochem.

[B15] Blatt J, White C, Dienes S, Friedman H, Foley T (1984). Production of an insulin-like growth factor by osteosarcoma. Biochem Biophysics Res Commun.

[B16] Yang D, Rogler CE (1991). Analysis of insulin-like growth factor II (IGF II) expression in neoplastic nodules and hepatocellular carcinoma of woodchucks utilizing in situ hybridization and immunohistochemistry. Carcinogenesis (Lond).

[B17] Modan-Moss D, Janicot M, Mclenithan JC, Lane MD, Casella SJ (1998). Expression and Function of insulin-like growth factor I hybrid receptors during differentiation of 3T3-L1 preadipocytes. Biochem J.

[B18] Faber E (1980). The sequential analysis of liver cancer induction. Biophys Biochim Acta.

[B19] Bannasch P, Zerban H, Hacker HJ, Jones TC, Popp J, Mohr U (1997). Foci of altered hepatocytes, rat. Monogrpahs on pathology of laboratory animals Digestive system.

[B20] Su Q, Benner A, Hofmann WJ, Otto G, Pichlmayr R, Bannasch P (1997). Human hepatic preneoplasia : phenotypes and proliferation kinetics of foci and nodules of altered hepatocytes and their relationship to liver cell dysplasia. Virchows Arch.

[B21] Bannasch P, Boyer JL, Ockner RK (1996). Pathogensis of hepatocellular carcinoma : sequential cellular, molecular and metabolic changes. Progress in liver disease.

[B22] Fiorentino M, Grigioni WF, Baccarini P, Errico D, De Mitri MS, Pisi E, Mancini AM (1994). Different in situ expression of insulin-like-growth factor type II in hepatocellular carcinoma : an in situ hybridization and immunohistochemical study. Diagn Mol Pathol.

[B23] Pence CB (1991). Dietary Selenium and anti-oxidant status; toxic effects of 12 demethyl hydrazine in rats. J Nut.

[B24] Mukherjee B, Basu M, Chaterjee M (2001). Effect of selenomethionine on N-methyl nitroso guanidine-induced colonic aberrant crypt foci in rats. Europian Journal of Cancer Prevention.

[B25] Bannasch P (1968). The cytoplasm of hepatocytes during carcinogensis. Recent Results in Cancer Research.

[B26] Bannasch P, Jahn UR, Hacker HJ, Hofmann W, Pichlmayr R, Otto G (1991). Focal hepatic glycogenosis: a putative preneoplastic lesion associated with neoplasia and cirrhosis in explanted human livers. Int J of Oncology.

[B27] Yang D, Alt E, Rogler CE (1993). Coordinate expression of N-myc2 and insulin-like growth factor II in precancerous altered hepatic foci in woodchuck hepatis virus carriers. Cancer Res.

[B28] Moreno FS, Rizzi MBSL, Dagil MLA (1991). Inhibitory effect of B-Carotene on preneoplastic lesions induced in Wiser rats by the resistant hepatocyte model. Carcinogenesis.

[B29] Braissant O, Wahli (1998). A simplified in situ hydridization protocol using non-radioactively labeled probes to detect abundant and rare mRNAs on tissue sections. Biochemica.

[B30] Enzmann H, Zerban H, Kopp-Schneider A, Loser E, Bannasch P (1995). Effects of low doses of N- Nitrosomorpholine on the development of early stages of hepatocarcinogenesis. Carcinogenesis.

[B31] Bannasch P, Blosch M, Zerban H (1981). Spongiosis hepatis: Specific changes of the perisinusoidal liver cells induced in rats by N- Nitrosomorpholine. Lab Invest.

[B32] Stroebel P, Mayer F, Zerban H, Bannasch P (1995). Neoplasm Deriving from the perisinusoidal (ito) cells in rat liver. Am J Path.

[B33] Amann G, Breitschopf H, Lassmann H, Suchanck Gm, Heniz-Erian P (1998). Cellular localization of insulin- like growth factor II mRNA in human fetus and the placenta : detection with a digoxigenin labeled cRNA probe and immunocytochemistry. Pediatr Res.

[B34] Nivet V, Hajduch E, Hainault I, Delattre J, Lavau M, Hanique B (1997). Sensitive northern blot by hybridization using digexigenin RNA probes for the mRNA detection of two gulcose transporter isoforms. Cell Mol Biol.

[B35] Sohda T, Oka Y, Iwata K, Gunn J, Kamimura S, Shijo H, Okumura M, Yun K (1997). Co-localization of insulin-like growth factor II and the proliferation marker-MIBI in hepatocellular carcinoma cells. J Clin Pathol.

[B36] Cariani E, Lasserre C, Seurin D, Hamelin B, Kemeny F, Franco D, Czech MP, Allurich A, Brechot C (1988). Differential expression of insulin-like-growth factor II mRNA in human primary liver cancers, benign tumors and liver cirrhosis. Cancer Res.

[B37] Norstedt GA, Levinewit A, Moller C, Eriksson LC, Andersson G (1988). Expression of insulin-like-growth factor I (IGF I) and IGF II mRNA during hepatic development, proliferation and carcinogensis in rat. Carcinogensis.

[B38] Lin YZ, Ding L, Chen JY (1993). Expression of IGF II and its relation to the differentiation of preneoplastic hepatocytes in rats. Chung-hua-Ping-Li-Hsuch-Tsa-Chih.

[B39] Chung CK, Antoniades HN (1992). Expression of c-cis/platelet-derived growth factor β, insluin-like growth factor I, and transforming growth factor alpha messenger RNAs and their respective receptor messenger RNAs in primary human gastric carcinomas : in vivo studies with in situ hybridization and immunocytochemsitry. Cancer Res.

[B40] Wang YZ, Wong YC (1998). Sex hormone-induced prostatic carcinogenesis in the noble rat : The role of insulin-like-growth factor-I (IGF I) and vascular endothelial growth factor (VEGF) in the development of prostate cancer. Prostate.

[B41] Dietrich DR, Candrian R, Marsman DS, Popp JA, Kanfmann WK, Swenberg JA (1994). Retrospective assessment of liver cell proliferation via PCA: a comparison with tritiated thymidine. Cancer Letter.

[B42] Aihara T, Noguchi S, Miyoshi Y, Sasaki Y, Nakamura Y, Monden M, Imaoka S (1998). Allelic imbalance of insulin-like-growth factor II gene expression in cancer in cancerous and precancerous lesions of the liver. Hepatology.

